# Lipopolysaccharide Affects the Proliferation and Glucose Metabolism of Cervical Cancer Cells Through the FRA1/MDM2/p53 Pathway

**DOI:** 10.7150/ijms.47360

**Published:** 2021-01-01

**Authors:** Xiaoyan Jiang, Jing Yuan, Yingyu Dou, Da Zeng, Songshu Xiao

**Affiliations:** 1 Department of Gynecology and Obstetrics, The Third Xiangya Hospital of Central South University, Changsha 410013, China.; 2 Department of Obstetrics, The People's Hospital of Qijiang District, Chongqing 401420, China.

**Keywords:** cervical cancer, FRA1, LPS, metabolism

## Abstract

Previous studies have found that LPS and FRA1 play opposite roles in cervical cancer. In addition, LPS functions by regulating the expression of FRA1 in many disease models, but there is currently no study of their relationship in the energy metabolism of tumor cells. This study, therefore, investigates the effects of LPS on FRA1-mediated glucose metabolism and the possible mechanisms it may have in cervical cancer cells. We constructed FRA1 stable overexpressing/ empty vector cervical cancer cell lines, where glucose consumption, the level of lactic acid production and the expression of energy metabolism related molecules were detected under the stimulation of LPS. At the same time, the changes in proliferation ability of cervical cancer cells were detected. We discovered that LPS promotes glucose consumption, lactic acid production, pentose phosphate bypass, and inhibits aerobic oxidation, by inhibiting the expression of FRA1; and that LPS promotes the growth of cervical cancer cells. Our results indicate that LPS affects the proliferation and glucose metabolism of cervical cancer cells through the FRA1/MDM2/p53 pathway.

## Introduction

According to the latest global cancer statistics 1, in 2018 there were approximately 569,847 new cases and 311,365 deaths from cervical cancer, which effectively ranks the disease fourth for both incidence (6.6%) and mortality (7.5%) amongst women. These staggering statistics, showing that cervical cancer not only threatens women's health but is a significant risk to life as well, therefore, give impetus to explore further the pathogenesis of cervical cancer and improve therapeutic outcomes.

Sustained rapid proliferation and abnormal energy metabolism are prominent features of tumor cells; features that potentially indicate the direction of cancer therapy 2. For nutritional and energy needs in support of their rapid proliferation, tumor cells use the process of glycolysis as their main source of energy, even when an oxygen supply is sufficient; in this circumstance, the process is termed aerobic glycolysis which is also known as the “Warburg effect”. Studies have found that the process of aerobic glycolysis in tumor cells is closely related to changes in their own genes. The oncogene *MYC* can up-regulate the level of lactate dehydrogenase A (LDHA), leading to an increase in lactic acid (LA) production. It can also promote the transcription of glucose transporter 1 (GLUT1), hexokinase 2, phosphor-fructose kinase, and increase cell glucose uptake and pyruvate production. 3. Mutations in *K-RAS* can increase the expression of GLUT1 as well as the uptake of glucose, enhance glycolysis and lactic acid production, and affect the occurrence and development of tumors 4.

FOS related antigen-1 (FRA1) is a member of the FOS family (c-FOS, FOS B, FRA1 and FRA2) 5. The FOS protein family can bind with the JUN family (c-JUN, JUN B and JUN D) through a leucine zipper domain to form the transcription factor complex AP-1, which plays an important role in embryonic development, bone formation and tumor progression. AP-1 binds to a specific sequence on the promoter of the target gene (phorbol ester response element, AP-1 site), thereby regulating the transcription of the target gene and participating in the proliferation, differentiation, invasion, angiogenesis, apoptosis and metastasis of tumor cells 6. We have previously reviewed the important role of FRA1 in the development of tumorigenesis 7. Also, we used flow cytometry, qRT-PCR and western blotting to confirm that FRA1 is down-regulated in cervical cancer tissues and regulates apoptosis of cervical cancer cells. Our results also showed that the regulation of FRA1 on cervical cancer is associated with p53 8.

Lipopolysaccharide (LPS) is an important component of the cell wall of gram-negative bacteria. Previous studies on LPS mainly focused on inflammatory response, but in recent years, more and more studies have found that LPS can regulate the proliferation and invasion ability of tumor cells, including breast cancer 9, pancreatic cancer 10, glioblastoma 11,12, astrocytoma 13, and promote the epithelial mesenchymal transformation (EMT) of liver cancer cells 10,14. These works demonstrate that a correlation exists between inflammation and tumorigenesis. LPS regulates tumor cells mainly through binding with toll-like receptor 4 (TLR4), and then activates the PKC, MAPKs, JNK and ERK1/2 signaling pathways, thereby affecting the activity of the transcription factors AP-1 and NF-κB 15. Recently, it was found that LPS can enhance the expression of HIF-1α in cervical cancer cells by binding to TLR4 and triggering reactive oxygen species (ROS) and NADPH, suggesting that LPS has an effect on the energy metabolism of cervical cancer cells 16, however, its exact mechanism remains unclear.

Previous studies have shown a relationship between LPS and FRA1. For example, in acute lung injuries, LPS promotes FRA1 expression in alveolar macrophages 17. Similarly, in osteoporosis, LPS can also promote the expression of FRA1 in osteoclasts 18. However, the role of LPS in FRA1-mediated energy metabolism of cervical cancer cells has hitherto not been studied. Hence, in this study FRA1 stable overexpressing/empty cervical cancer cell lines were constructed to detect the glucose metabolism-related products, such as glucose consumption and lactic acid production under the stimulation of LPS. The results show that by inhibiting the FRA1/MDM2/p53 pathway, LPS can inhibit the aerobic oxidation, promote the glycolysis and the pentose phosphate pathway (PPP) as well as promote the malignant process of cervical cancer cells.

## Materials and methods

### Cell culture

The human cervical cancer cell lines Caski and Hela were cultured in RPMI-1640 supplemented with 10% fetal bovine serum (FBS) (Gibco, USA), penicillin (100 U/ml) and streptomycin (100 μg/ml) at 37˚C and 95% humidity in the presence of 5% CO2.

### Construction of the stable FRA1-overexpressing cell lines

The LV5-FRA1 lentivirus was purchased from Suzhou GenePharma (Jiangsu, China). The plasmid was cut with NotI/BamHI and ligated by T4 DNA ligase with gene encoding FRA1. The fusion sequence was verified through DNA sequencing. The empty LV5 vector was used as a negative control. To establish the stable FRA1-overexpressing Caski and Hela cell lines, LV5-FRA1 or an empty LV5-vector (10ul) was added into Caski and Hela cells at a density of 50-60%, together with the polybrene transfection reagent (1ul). After 48 hours, screening was performed using puromycin. Transduction efficiency was measured using qRT-PCR and western blotting experiments.

### RNA extraction and qRT-PCR

LPS (50 ug/ml) was added to Caski-FRA1/Caski-empty vector and Hela-FRA1/Hela-empty vector cells for 24h at 37 ˚C. The total RNA was extracted from cultured cells using the TRIzol reagent. RNA was reverse transcribed into complementary DNA (cDNA) using a HiScript II Q RT SuperMix for qPCR (+gDNA wiper) kit (Vazyme, Nanjing, China). The qRT-PCR technique was performed using a ChamQTM SYBR® qPCR Master Mix kit (Vazyme, Nanjing, China). The primer sequences used for qRT-PCR were as follows: FRA1 forward (CGAAGGCCTTGTGAACAGAT) and reverse (CTTCTGCTTCTGCAGCTCCT); GAPDH forward (CGACCACTTTGTCAAGCTCA) and reverse (ACTGAGTGTGGCAGGGACTC).

### Western blotting

LPS (50 ug/ml) was added to Caski-FRA1/Caski-empty vector and Hela-FRA1/Hela-empty vector cells for 24h at 37 ˚C. Cells were lysed with RIPA lysate buffer containing 1% protease inhibitor. The protein concentration was measured using the BCA protein quantitative kit (Lianke biotech, China). Equivalent quantities (30-50 μg) of protein were separated in 10% SDS‑PAGE gels and electroblotted onto PVDF membranes (Millipore, USA). The membranes were blocked using Tris-buffered saline containing 5% non-fat milk. After that, the primary antibody was incubated overnight at 4 °C. The primary antibodies used in this study were as follows: FRA1, SCO2, COX2, G6PD (ImmunoWay Biotechnology Co); LDHA, MDM2, p53 (Wuhan Boster Biological Technology, Ltd.); GLUT1 (Abcam). After three washes, the secondary antibody (Goat Anti-Rabbit Ig(H+L) HRP or Goat Anti-Mouse Ig(H+L) HRP, Affinity, USA) was incubated at 37 ° C for 1h. After another three washes, the expression of protein was detected with a LuminataTM Forte Western HRP Substrate luminescence solution (Millipore, USA). Then β-actin (Santa Cruz Biotechnology, Inc.) was used as a loading control. The quantification of band intensity was performed by using Image J (National Institutes of Health, USA).

### CCK-8 cell proliferation assay

LPS (50 ug/ml) was added to Caski-FRA1/Caski-empty vector and Hela-FRA1/Hela-empty vector cells (1 x10^3^ cell/well) for 24h at 37 ˚C. Subsequently, 20ul CCK-8 was added to the cells for 2h at 37˚C in 5% CO2. The SoftMax Pro 6.4 microplate reader (Beckman Coulter, USA) was used for detecting the absorbance at 450 nm. The above steps were repeated at the same time every day.

### Colony formation assay

LPS (50 ug/ml) was added to Caski-FRA1/Caski-empty vector and Hela-FRA1/Hela-empty vector cells (1 x10^3^ cell/well) for 12 days at 37 ˚C, until visible clones grow in the 6-well plate. After washing 3 times with PBS, cells were fixed with 4% paraformaldehyde for 30 minutes, and then stained with 0.1% crystal violet solution. After washing the background with PBS and leaving plates to dry, pictures were then taken with a camera. Image J was used to quantify the number of clones.

### Glucose consumption assay

Fresh culture medium was replaced when the density of Caski-FRA1/Caski-empty vector and Hela-FRA1/Hela-empty vector cells in the 6-well plate reached 50-60%, and LPS (50 ug/ml) was added to the cells for 24h at 37°C. The extracellular medium was collected and the glucose levels in the culture medium were measured according to the protocol as defined in the commercial Glucose Assay Kit (Jiancheng, Nanjing, China). The absorbance at 505 nm of the Glucose level was measured using a SoftMax Pro 6.4 microplate reader (Beckman Coulter, USA).

### Lactic acid assay

Fresh culture medium was replaced when the density of Caski-FRA1/Caski-empty vector and Hela-FRA1/Hela-empty vector cells in the 6-well plate reached 50-60%, and LPS (50 ug/ml) was added to the cells for 24h at 37°C. The cell supernatant was collected and the lactate levels in the cells were measured according to the protocol as defined in the Lactic Acid Assay Kit (Jiancheng, Nanjing, China). The absorbance at 530 nm of lactate levels were measured using a SoftMax Pro 6.4 microplate reader (Beckman Coulter, USA).

### ATP measurements

Fresh culture medium was replaced when the density of Caski-FRA1/Caski- empty vector and Hela-FRA1/Hela-empty vector cells in the 6-well plate reached 50-60%, and LPS (50 ug/ml) was added to the cells for 24h at 37°C. Whole cell lysates were collected and the ATP levels in the cells were measured according to the protocol as defined in the Enhanced ATP Content Detection Kit (Beyotime, Shanghai, China). The ATP levels were measured using a SoftMax Pro 6.4 microplate reader (Beckman Coulter, USA). Cell concentration was measured to normalize the level of ATP.

### 6-phosphate glucose dehydrogenase (G6PD) assay

Fresh culture medium was replaced when the density of Caski-FRA1/Caski-empty vector and Hela-FRA1/Hela-empty vector cells in the 6-well plate reached 50-60%, and LPS (50 ug/ml) was added to the cells for 24h at 37°C. Whole cell lysates were collected and the G6PD levels in the cells were measured according to the protocol as defined in the G6PD Activity Assay Kit (solarbio, Beijing, China). The absorbance (340 nm) at 0s and 300s at 37℃ were measured using a SoftMax Pro 6.4 microplate reader (Beckman Coulter, USA). Cell concentration was measured to normalize the level of G6PD.

### Statistical analysis

The data were processed using the GraphPad Prism software V7.0 such that each data point was translated into its mean + standard deviation values from three independent repetitions of the experiment. The student's t-test was used to analyze the statistical comparisons between two groups. *P*<0.05 was considered statistically significant.

## Results

### LPS affects the FRA1 expression of cervical cancer cells

**Figure 1:**
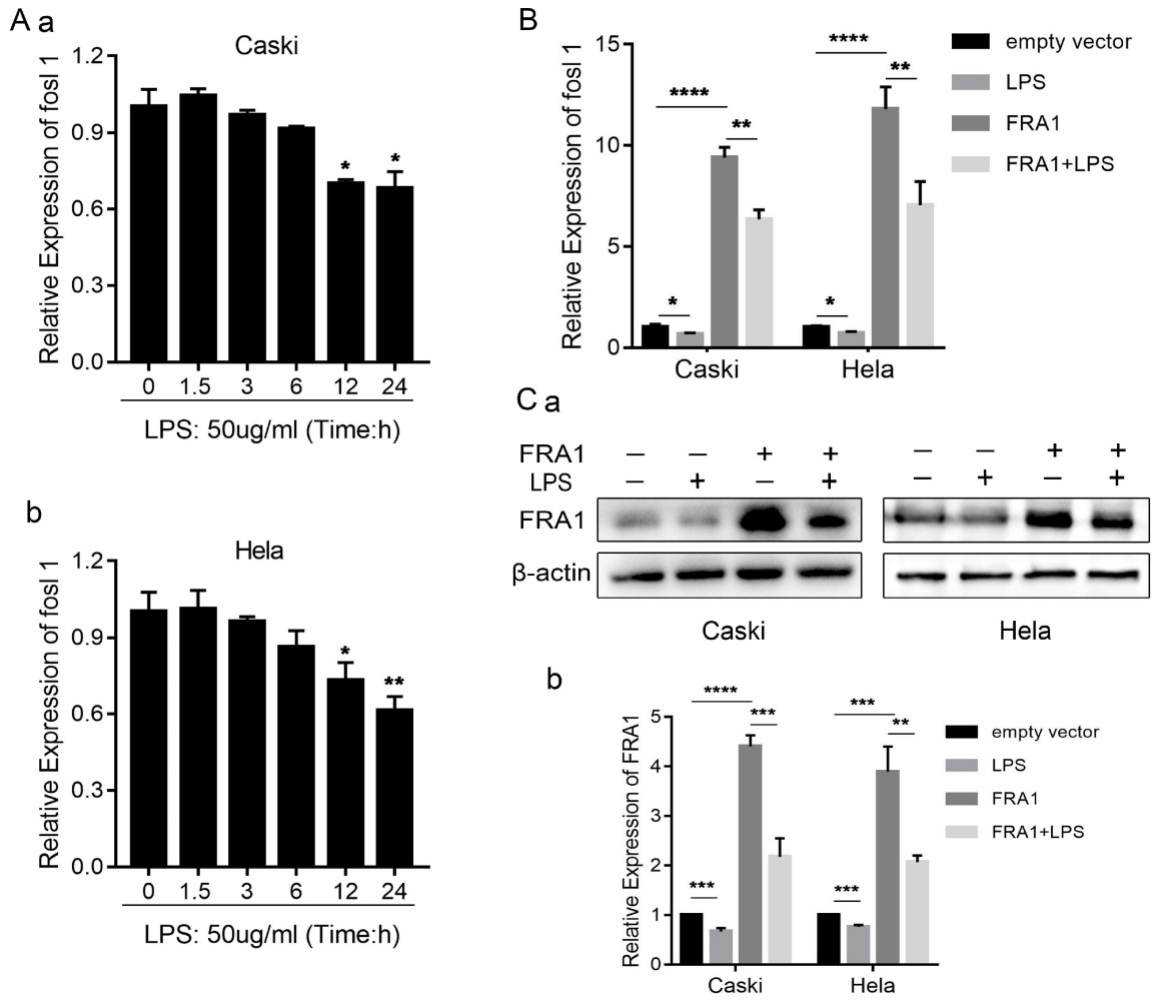
LPS affects FRA1 expression in cervical cancer cells: (A) qRT-PCR analysis of *FOSL1* mRNA levels in Caski and Hela cell lines at different time points after LPS (50ug/ml). (B) qRT-PCR analysis of *FOSL1* mRNA levels in Caski-FRA1/Caski-empty vector and Hela-FRA1/Hela-empty vector cells after LPS (50ug/ml). (C) (a) Western blotting analysis of FRA1 protein levels in Caski-FRA1/Caski-empty vector and Hela-FRA1/Hela-empty vector cells after LPS (50ug/ml). (b) Represent relative expression levels of FRA1. *P<0.05, **P<0.01, ***P<0.001, ****P<0.0001.

In order to determine if LPS can affect the FRA1 level of cervical cancer cells, we examined the changes of *FOSL1* mRNA at different time points after adding LPS (50ug/ml). The results show that LPS has no significant effect on FRA1 in the early stage, but after 12 hours FRA1 was significantly down-regulated and the results were statistically different. This indicates that LPS can affect the FRA1 expression level of cervical cancer cells in a time depended manner (Figure 1A).

To investigate further the relationship between LPS and FRA1, we established FRA1 stable overexpressing cervical cell lines (Caski-FRA1/Hela-FRA1) and corresponding blank control cell lines (Caski-empty vector/Hela-empty vector) through lentiviral infection. The Caski-FRA1/Caski-empty vector and Hela-FRA1/Hela-empty vector cells were treated with LPS for 24 hours. The results of qRT-PCR and western blotting tests show that LPS can indeed down-regulate the expression level of FRA1 in cervical cancer cells (Figure 1B, C).

### LPS negatively regulates the effects of FRA1 on glucose metabolism of cervical cancer cells

**Figure 2:**
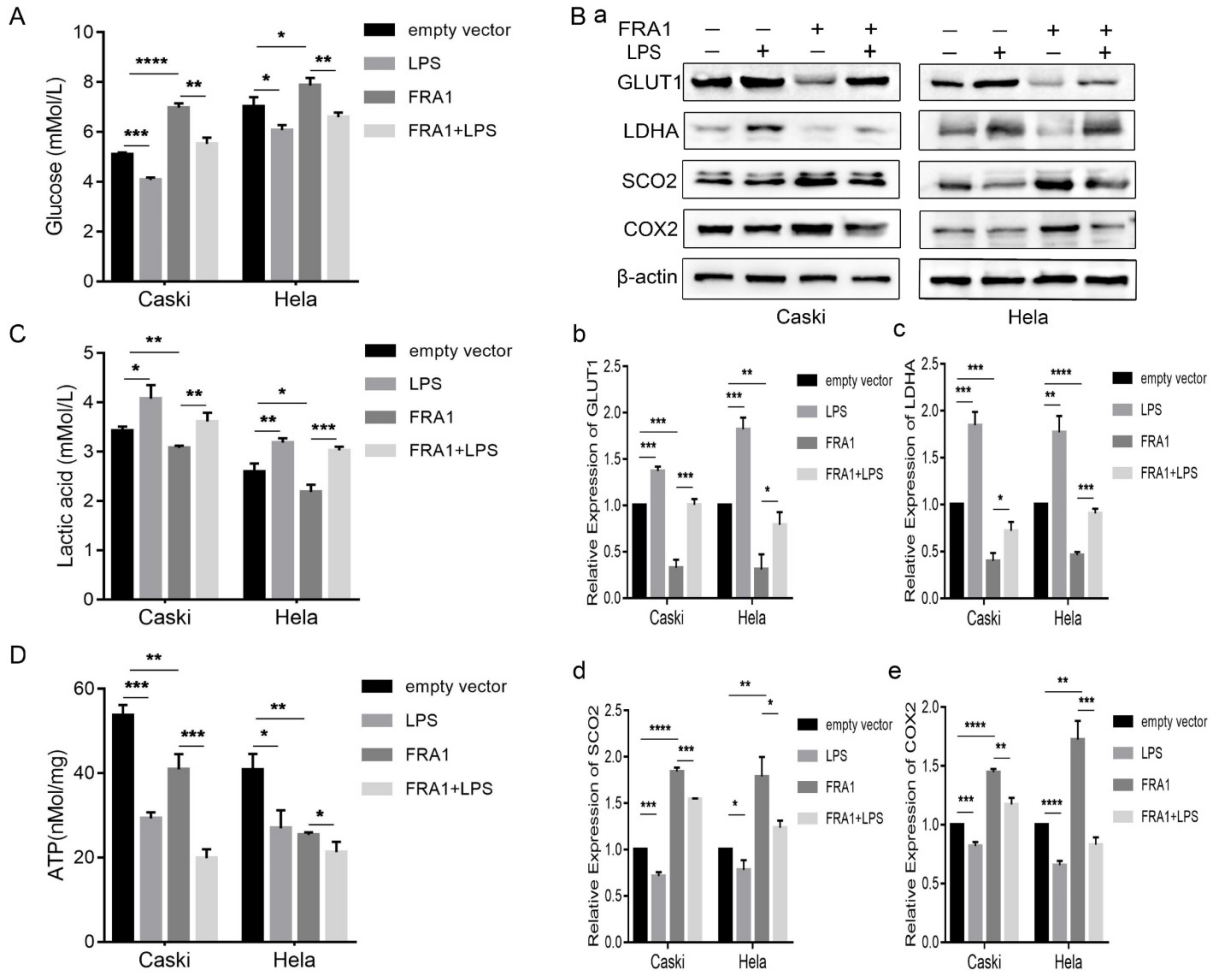
LPS negatively regulates FRA1-mediated glucose metabolism in cervical cancer cells: (A) Glucose content in extracellular medium of Caski-FRA1/Caski-empty vector and Hela-FRA1/Hela-empty vector cells after LPS (50ug/ml). (B) (a) Western blotting analysis of GLUT1, LDHA, SCO2 and COX2 protein levels in Caski-FRA1/Caski-empty vector and Hela-FRA1/Hela-empty vector cells after LPS (50ug/ml). (b-e) Represent relative expression levels of GLUT1, LDHA, SCO2, COX2. (C) Lactic acid production of Caski-FRA1/Caski-empty vector and Hela-FRA1/Hela-empty vector cells after LPS (50ug/ml). (D) Intracellular ATP of Caski-FRA1/Caski-empty vector and Hela-FRA1/Hela-empty vector cells after LPS (50ug/ml). *P<0.05, **P<0.01, ***P<0.001, ****P<0.0001.

As an important source of glucose metabolism, glucose consumption is closely related to the metabolic level of cells. We first tested the glucose content in an extracellular medium, and found that the glucose content in the FRA1-overexpressing group was higher than that of the corresponding empty vector group. However, when LPS was added, the glucose content decreased significantly (Figure 2A). This observation shows that FRA1 can reduce glucose consumption, while LPS can increase glucose consumption in cervical cancer cells.

GLUT1 is an important transporter for glucose to enter a cell from the extracellular. Detection of its expression level further confirms that FRA1 reduces glucose entry into a cell, while LPS promotes glucose entry into a cell (Figure 2B).

As a product of glycolysis, the content of LA can thus reflect the level of glycolysis and, furthermore, it should be noted that lactate dehydrogenase is the key enzyme in the production of LA from pyruvate. The lactate and LDHA levels were also measured in eight groups of cells. The results show that FRA1 can inhibit the expression of LDHA and the production of LA in cervical cancer cells, while LPS negatively regulated the inhibitory effect of FRA1 on glycolysis (Figure 2B, C).

Synthesis of cytochrome C oxidase 2 (SCO2) and Cytochrome C oxidase subunit II (COX2) are important substances involved in aerobic oxidation. We examined the expression levels of them in four groups of cells. The results show that FRA1 promoted their expression, but after the addition of LPS, this pro-expression was significantly down-regulated (Figure 2B). This indicates that FRA1 can promote aerobic oxidation of cells, and LPS reversely regulates the action of FRA1.

Subsequently, we also directly quantified the ATP content in eight groups of cells. The results show that ATP content in the FRA1-overexpressing group was lower than that in the corresponding empty vector group, and the addition of LPS did not increase the ATP content, but further down-regulated it (Figure 2D). We suspect this is because the direct detection of ATP content at a certain point is not sufficient to reflect the dynamic process of ATP production.

**Figure 3:**
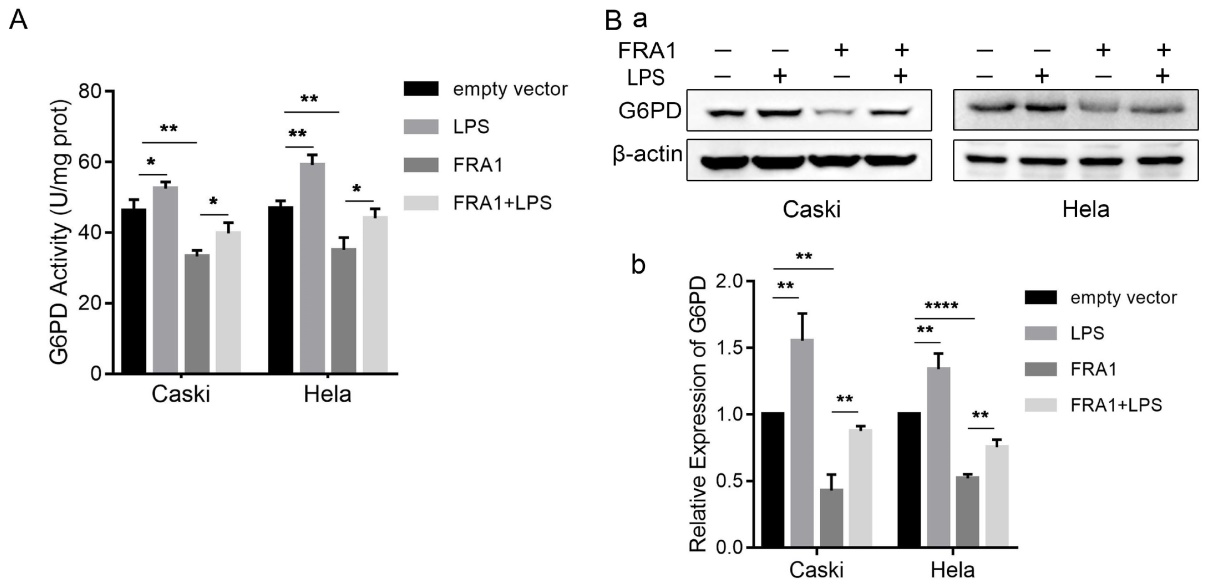
LPS negatively regulates FRA1-mediated PPP in cervical cancer cells: (A) G6PD activity of Caski-FRA1/Caski-empty vector and Hela-FRA1/Hela-empty vector cells after LPS (50ug/ml). (B) (a) Western blotting analysis of G6PD protein levels in Caski-FRA1/Caski-empty vector and Hela-FRA1/Hela-empty vector cells after LPS (50ug/ml). (b) Represent relative expression levels of G6PD. *P<0.05, **P<0.01, ****P<0.0001.

PPP is an important branch of glucose catabolism. The main process involved is that glucose-6-phosphate produces NADPH and 5-phosphate ribose under the catalyzation of G6PD. Therefore, we also examined the protein expression level and enzyme activity of G6PD in cells. The results show that FRA1 can simultaneously inhibit the expression of G6PD and its enzymatic activity, but this inhibition was significantly attenuated after the addition of LPS (Figure 3A, B).

### LPS negatively regulates FRA1-mediated growth inhibition in cervical cancer cells

**Figure 4:**
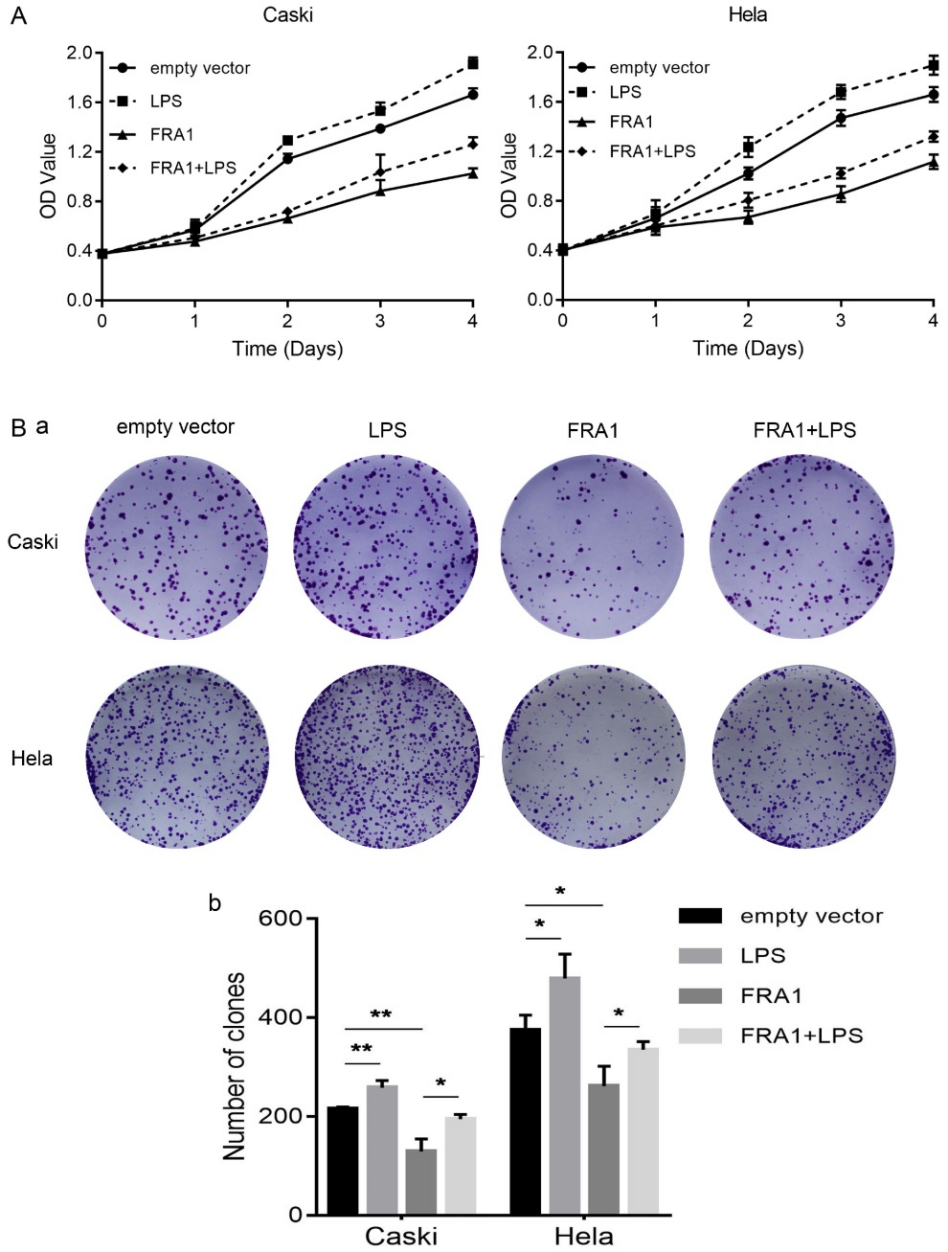
LPS negatively regulates FRA1-mediated growth inhibition in cervical cancer cells: (A) CCK-8 assay was used to detect the proliferation of Caski-FRA1/Caski-empty vector and Hela-FRA1/Hela-empty vector cells after LPS (50ug/ml). (B) (a) colony formation assay was used to detect the proliferation of Caski-FRA1/Caski-empty vector and Hela-FRA1/Hela-empty vector cells after LPS (50ug/ml). (b) Number of clones. *P<0.05, **P<0.01.

In order to observe, more intuitively whether changes in glucose metabolism affect the growth of cervical cancer cells, we also directly detected the proliferation of eight groups of cells through the CCK-8 and colony formation assays. The results show that FRA1 inhibited the growth of cervical cancer cells, but this inhibition was attenuated after the addition of LPS (Figure 4A, B).

### The effect of LPS and FRA1 on cervical cancer is related to MDM2/p53

**Figure 5:**
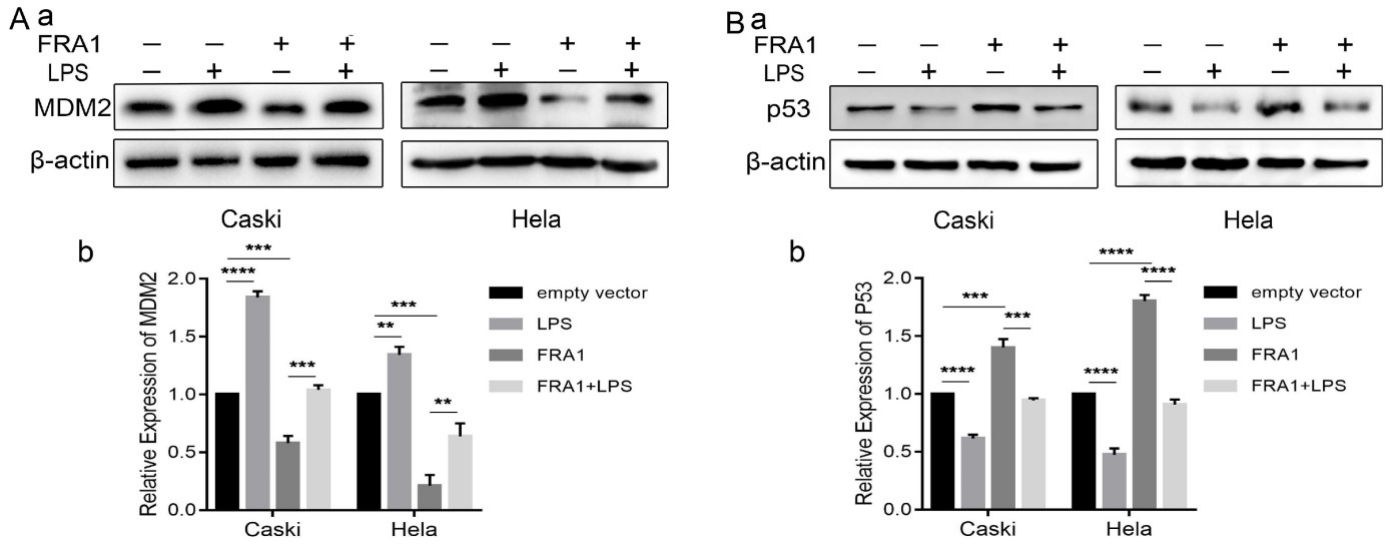
The effect of LPS and FRA1 on cervical cancer is related to MDM2/p53: (A) and (B) (a) Western blotting analysis of MDM2 and p53 protein levels in Caski-FRA1/Caski-empty vector and Hela-FRA1/Hela-empty vector cells after LPS (50ug/ml). (b) Represent relative expression levels of MDM2 and p53. **P<0.01, ***P<0.001, ****P<0.0001.

**Figure 6:**
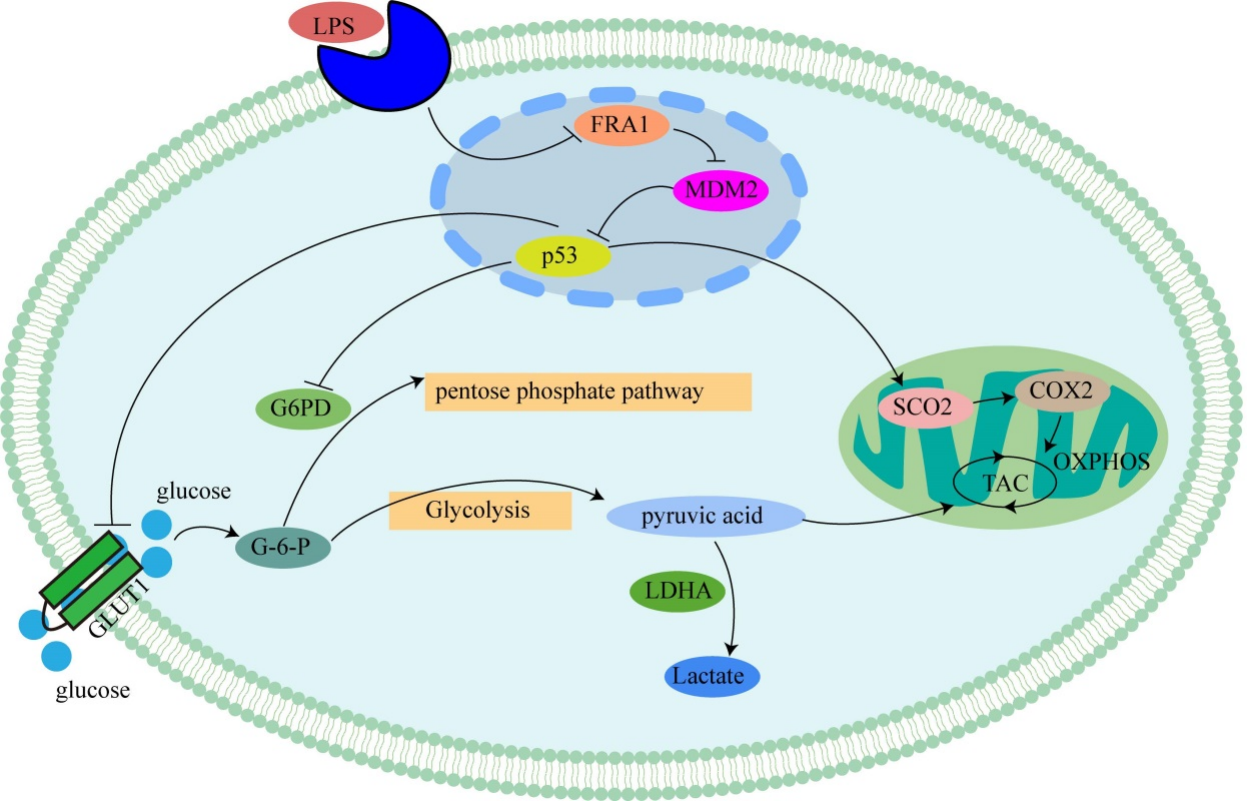
Mechanism of LPS affecting glucose metabolism in cervical cancer cells through FRA1: LPS inhibits p53 by inhibiting the expression of FRA1, which regulates the levels of glycolysis and aerobic oxidation by affecting downstream GLUT1, G6PD and SCO2.

Our previous studies 8 have shown that the effect of FRA1 on cervical cancer is related to MDM2/p53, which, therefore, prompted an examination in this study of the expression levels of MDM2 and p53. The results show that FRA1 can promote the level of p53 by down-regulating the expression of MDM2. After the addition of LPS, however, MDM2 expression was up-regulated, resulting in the down-regulation of p53 expression (Figure 5A, B). We propose that LPS can regulate the MDM2/p53 pathway by acting on FRA1, thereby affecting glucose metabolism and, therefore, the growth of cervical cancer cells (Figure 6).

## Discussion

Previous studies have shown that in cervical cancer, FRA1 expression decreases with the increase of malignancy and plays a role in inhibiting the occurrence and development of cervical cancer 19. In contrast, LPS can bind to its receptor TLR4 and promote the proliferation of cervical cancer cells through the MyD88 and NF-κB pathways 20. Based on their respective functions in cervical cancer cells, and the importance of changes in energy metabolism in tumor cells, our experiments investigated whether LPS affects the FRA1-mediated glucose metabolism in cervical cancer cells.

We first tested the LPS influence on FRA1 expression, the results show that LPS can down-regulate FRA1 expression in cervical cancer cells. Then, we examined the glucose content in extracellular medium and found that FRA1 can reduce glucose consumption, but LPS significantly increased demand for glucose in cervical cancer cells. In tumor cells, glucose is mainly used for glycolysis to provide energy, whilst cutting down the level of oxidative phosphorylation (OXPHOS). Therefore, we directly measured the content of the glycolytic product LA and the expression of SCO2 and COX2, which are closely related to OXPHOS. It was found that LA production in FRA1-overexpressing cells is less than that in the corresponding empty vector cells, but that LA production increased after adding LPS. Meanwhile, the expression levels of SCO2 and COX2 show an opposite trend. In addition, we also tested the GLUT1 and LDHA expression and found that LPS can negatively regulate FRA1-induced down-regulation of them, which accords with the glucose consumption and the level of the LA generated. Moreover, we directly measured the ATP content in eight groups of cervical cancer cells. The results show less ATP in the FRA1-overexpressiing groups. To our surprise, after adding LPS, the ATP content did not rise correspondingly as expected, but even became less. By reviewing the data, we learned that although glucose produces less ATP through glycolysis than OXPHOS, tumor cells can increase the speed of glycolysis by up-regulating the glucose transporter, and increase the speed of ATP production to meet energy requirements 2. Therefore, we believe that if we can dynamically monitor the rate of ATP production in living cells, we can more appropriately reflect the energy generation. Unfortunately, due to the limitations of our experimental conditions, especially in relation to experimental facilities, we could not dynamically monitor ATP production in living cells.

One of the most important processes of the Warburg effect is the enhancement of the PPP bypass. A large amount of glucose is decomposed into 5-phosphate ribose and NADPH by PPP, providing precursors for intracellular synthesis of nucleic acids and fatty acids 21. In this study, we examined the expression of G6PD, a key enzyme in the PPP bypass, and found it was lower in cells with higher levels of FRA1 expression, and the expression of G6PD was significantly increased after LPS. The detection of G6PD enzyme activity is consistent with the protein expression level, suggesting that FRA1 can inhibit the PPP bypass of cervical cancer cells, while LPS can negatively regulate the inhibition of FRA1 and restore the PPP bypass level of cervical cancer cells. However, further detection of its metabolites such as NADPH and 5-phosphate ribose are required to figure out the detailed ways of the influence of LPS on PPP.

We also used CCK-8 and colony formation assays to detect directly the proliferation of cervical cancer cells. The results show that FRA1 can inhibit the growth of cervical cancer cells, while LPS decreases FRA1-induced inhibition. This result is consistent with the results of previous research 20,22, and also indicates that changes in glucose metabolism can directly affect the growth state of cells.

Our previous study 8 concluded that the role of FRA1 in cervical cancer is associated with p53. Moreover, in this study, we further demonstrated that LPS can negatively regulate FRA1-mediated changes in MDM2/p53 expression, which then affects glucose metabolism. As one of the most widely studied tumor suppressor genes, p53 plays an important role in regulating the energy metabolism of cells. P53 regulates the balance between mitochondrial respiration and glycolysis and determines how ATP is produced. P53 can directly inhibit GLUT1 and GLUT4 and indirectly inhibit the expression of GLUT3 23, inhibit glucose consumption, increase both the TP53-induced glycolysis and apoptosis regulator (TIGAR) to reduce the production of fructose-2,6-diphosphate. Also, p53 induces glutaminase expression and reduces phosphoglucomutase expression, resulting in the inhibition of glycolysis, reducing the role of energy production, and ultimately inhibiting tumor progression 24. Furthermore, wild-type p53 enhances the activity of SCO2, catalyzes the conversion of pyruvate to acetyl-CoA for the expression of pyruvate dehydrogenase 1 (PDH1), and inhibits tumor production by promoting OXPHOS 25. In addition, studies have found that p53 can also regulate G6PD activity and the PI3K-AKT-mTOR pathway to inhibit PPP and NADPH production, thereby inhibiting the production of fatty acids from glucose 26. Our study is consistent with the role of p53 as described above.

In conclusion, our study explores the role of LPS and FRA1 in cervical cancer cells from the perspective of glucose metabolism. The results indicate that LPS affects glucose metabolism in cervical cancer cells through the FRA1/MDM2/p53 pathway, thus affecting the proliferation of cervical cancer. This work indicates the potential for using FRA1 in therapies for cervical cancer, as well as providing theoretical support for the development of therapies targeting energy metabolism in cervical cancer cells.

## Abbreviations

FRA1: FOS related antigen-1
LPS: lipopolysaccharides
qRT-PCR: quantitative real-time polymerase chain reaction
TLR-4: Toll-like receptor 4
CCK-8: Cell Proliferation and Cytotoxicity Assay Kit
